# Idiopathic pulmonary fibrosis associated with pulmonary vein thrombosis: a case report

**DOI:** 10.1186/1757-1626-2-9156

**Published:** 2009-12-07

**Authors:** Raquel A Cavaco, Sunny Kaul, Timothy Chapman, Romina Casaretti, Barbara Philips, Andrew Rhodes, Michael R Grounds

**Affiliations:** 1 Department of Internal Medicine, Centro Hospitalar Lisboa Norte, Lisbon, Portugal; 2 Department of Respiratory Medicine, Kings College Hospital, London, UK; 3 Department of Respiratory Medicine, St George's Hospital, London, UK; 4 Department of Anaesthesiology, Department of Anaesthesia and Intensive Care Medicine, University of Udine, Udine, Italy; 5 Department of Anaesthesiology and Intensive Care, St George's Hospital, London, UK

## Abstract

**Background:**

Pulmonary vein thrombosis represents a potentially fatal disease. This syndrome may clinically mimic pulmonary embolism but has a different investigation strategy and prognosis. Pulmonary vein thrombosis is difficult to diagnose clinically and usually requires a combination of conventionally used diagnostic modalities.

**Case Presentation:**

The authors report a case of a 78-year-old previously healthy female presenting with collapse and shortness of breath. Serum biochemistry revealed acute kidney injury, positive D-dimmer's and increased C reactive protein. Chest radiography demonstrated volume loss in the right lung. The patient was started on antibiotics and also therapeutic doses of low molecular weight heparin. The working diagnosis included community acquired pneumonia & pulmonary embolism. A computed tomography pulmonary angiogram was performed to confirm the clinical suspicions of pulmonary embolism. This demonstrated a thrombus in the pulmonary vein, with associated fibrosis and volume loss of the right lower lobe. A subsequent thrombophilia screen revealed a positive lupus anticoagulant antibody and rheumatoid factor and also decreased anti thrombin III and protein C levels. The urine protein/creatinine ratio was found to be 553 mg/mmol.

**Conclusion:**

The diagnosis of this patient was therefore of idiopathic pulmonary fibrosis associated with pulmonary vein thrombosis. Whether or not the pulmonary vein thrombosis was a primary cause of the fibrosis or a consequence of it was unclear. There are few data on the management of pulmonary vein thrombosis, but anticoagulation, antibiotics, and, in cases of large pulmonary vein thrombosis, thrombectomy or pulmonary resection have been used.

## Introduction

Pulmonary vein thrombosis (PVT) is a rare but potentially life threatening condition. Its rare occurrence is due to a rich network of venous collateral vessels that drain the lung, however certain clinical conditions can lead to obstruction of the pulmonary veins and subsequent infarction [1,2]. Causes include: surgery involving veins such as lung transplantation or lobectomy; radiofrequency catheter ablation (RFCA) for atrial fibrillation; sclerosing mediastinitis; and certain primary or secondary tumours of the lung. Less common causes include: atrial myxoma; congenital pulmonary venous narrowing; and mitral stenosis with an obstructing left atrial clot [2-9]. PVT can presents in one of two fashions: acutely as pulmonary infarction with cough, dyspnoea and pleuritic chest pain or in a more insidious manner as progressive or recurrent pulmonary oedema and pulmonary fibrosis [1-3]. PVT is difficult to diagnose clinically, as reported associated signs and symptoms are non-specific, and usually requires a combination of conventional diagnostic modalities such as ventilation-perfusion scanning, pulmonary angiography, bronchoscopy, transthoracic echocardiography (TTE), trans-oesophageal echocardiography (TOE), computed tomography (CT) and, more recently, magnetic resonance imaging (MRI) [1,2,4,6,7,10-12]. Treatment of these patients should be determined on the basis of the obstructing pathological finding and can include antibiotic therapy, anticoagulation, thromboembolectomy and/or pulmonary resection [4,8,13-15]. We report a case of pulmonary vein thrombosis in a previous healthy patient presenting with collapse, shortness of breath and fibrosis of the right lung on a CT scan.

## Case presentation

**Figure 1:**
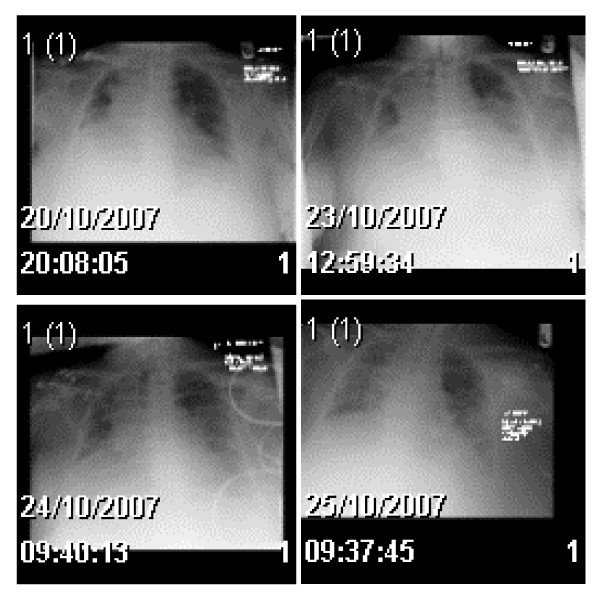
**Chest films of the 1st to the last day of staying in the Unit**. On the early film there is volume loss in the right lung. Subsequent films show the patient has had a left-sided central line introduced and more recently the patient has been intubated. There is air space shadowing throughout both lungs.

**Figure 2:**
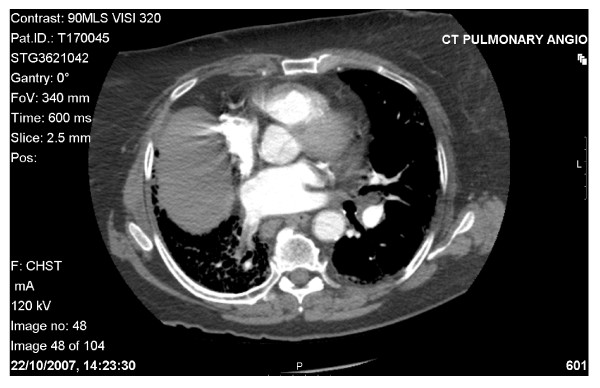
**CT pulmonary arteriography with venous phase imaging**. There's no evidence of pulmonary artery embolus but there is a thrombus seen in the right lower lobe pulmonary vein. There's evidence of a background of extensive right lung fibrosis and volume loss.

A white British, 78-year-old previously healthy female, non-smoker and with no known pulmonary disease, was admitted with collapse and shortness of breath with arterial oxygen saturations of haemoglobin of 80% on room air. The background included a history of persistent dry cough for 3 months without any other complaint or systemic symptoms. Her past medical history comprised of type 2 diabetes, increased uric acid and ongoing pain in the right knee. Her medication history consisted of gliclazide, allopurinol and diclofenac. The patient denied any recent travel or contact with animals (even pets). Physical examination revealed central cyanosis, tachypnoea, bilateral reduction in breath sounds and right-sided bronchial breathing. Cardiac examination was normal. There were no adenopathy noted. Arterial blood gases analysis on 5 litres of O2 per min showed mixed acidaemia. Serum biochemistry revealed raised urea (15.1 mmol/L) and creatinine (114 umol/L) levels consistent with acute kidney injury, positive D-dimmer's levels and increased CRP (48.2 mg/L). Chest radiography showed a loss of volume in the right lung (Figure 1) and an electrocardiogram demonstrated normal sinus rhythm with a heart rate of 94 bpm, left axis deviation and an old inferior myocardial infarction. At this time the patient was started on treatment for both a possible community acquired pneumonia (benzyl penicillin and clarythromycin) and for pulmonary thromboembolism (therapeutic doses of Dalteparin). The patient was stable for 48 hours but then her oxygen requirements increased progressively. Contrast-enhanced chest CT scan and CT pulmonary arteriography with venous phase imaging failed to demonstrate any evidence of pulmonary artery thrombus. However thrombus, with associated fibrosis and volume loss, was seen to affect one of the right lower lobe pulmonary veins (Figure 2). An echocardiogram was performed which showed a normal sized left ventricle with good function, normal aortic and mitral valves. The right heart although poorly visualized, appeared to be mildly enlarged with moderate function. The patient was started on methylprednisolone 1 g once a day and transferred to the intensive care unit (ICU).

The patient continued to deteriorate over the next 24 hours following admission to the ICU. She developed biochemical and electrocardiographic evidence of an acute anterior-lateral myocardial infarction and aspirin, clopidogrel and simvastatin were added to her anticoagulation therapy. Unfortunately the patient's oxygen requirements continued to increase and she was ventilated, initially supported by biphasic positive airway pressure (BIPAP) and then subsequently by invasive mechanical ventilation. Over the next eight days, despite maximal therapy she continued to deteriorate and died from multiple organ failure.

## Discussion

This case represents a form of pulmonary vein thrombosis presenting as a sub-acute disease with cough and dyspnoea but with radiological evidence of unilateral pulmonary fibrosis in a patient without previous known pulmonary disease. Throughout this patients admission we considered the possible primary cause of her PVT and whether the fibrosis was a causative event or consequence of the venous thrombosis. The only abnormal results from the thrombophilia screen were a slightly decreased anti thrombin III level (60 iu/dl - Normal > 75), a reduced protein C level (54 iu/dl - Normal > 70) and weakly positive lupic anticoagulant antibody (dTP, DRVVT) and rheumatoid factor. She had normal levels of immunoglobulin's (E, G, A, M), Complement C3 and C4, ECA and also alpha 1 anti-trypsin. Serology for Pneumococcus, Legionella, Aspergillus and Histoplasmosis were all negative. The urinary protein concentration was 5.75 g/dL with an urine protein/creatinine ratio of 553 mg/mmol (normal < 15), albumin 3.3 mg/L, normal eosinophil and triglycerides and ESR 36 (normal for her age). No serum or urinary paraprotein was detected. Little is known regarding the treatment of pulmonary vein thrombosis but the management of this syndrome seems dependent on the cause which is not always possible to identify [1,8]. In this case, therefore, treatment was directed according to the radiological findings, treating the thrombosis with therapeutic doses of low molecular weight heparin (LMWH) and trying to achieve the regression of pulmonary fibrosis with high doses of corticosteroids. Throughout the period of investigation, the patient was in the intensive care unit undergoing mechanical ventilation. She was cardiovascularly unstable and needed renal replacement therapy. Her unstable status precluded many investigations that would have been otherwise desirable to elucidate the underling aetiology. These tests would have included trans-oesophageal echocardiography, bronchoscopy and renal or open lung biopsy. Post mortem examination of the lungs indicated interstitial lung disease was present, although this was of little help in establishing a final diagnosis. Potential mechanisms leading to her pulmonary vein thrombosis include: venous pulmonary stasis due to pulmonary fibrosis; hypercoagulation state due to a nephritic syndrome; and catastrophic antiphospholipid syndrome.

## Conclusion

PVT has two main forms of presentation, acutely as pulmonary infarction or progressive as recurrent pulmonary oedema. The main causes include surgery to the lung, radiofrequency catheter ablation and certain tumours of the lung [2-5]. It should always be considered in patients with predisposing factors. Diagnostic confirmation is made with imaging studies, most frequently TOE and Chest CT scan [7,8,10,14,15].

Though currently uncommonly diagnosed, PVT diagnoses will potentially increase with the rising number of lung transplants, lobectomies and RFCA being performed but a high index of suspicion remains key to the diagnostic process. TOE may be advantageous in critically ill patients since the procedure in performed at the bedside and it can quantify the degree of obstruction and direct therapy appropriately. CT scans however are less invasive and require less highly trained personnel. MR imaging, particularly that performed with intravascular gadolinium injection, has been shown to reveal venous thrombosis [10,14]. No studies have been conducted regarding management of PVT, but anticoagulation, antibiotics, and, in cases of large PVT, thrombectomy or pulmonary resection in resectable tumours have been used [5,7,8,15].

## Consent

Written informed consent was obtained from the patient for publication of this case report and accompanying images. A copy of the written consent is available for review by the Editor-in-Chief of this journal.

## Competing interests

The authors declare that they have no competing interests.

## Authors' contributions

RC has made substantial contributions to acquisition and interpretation of data and wrote the manuscript. SK has been involved in drafting the manuscript and revising it critically for important intellectual content. TC has been involved in drafting the manuscript or revising it critically for important intellectual content. RC has been involved in drafting the manuscript or revising it critically for important intellectual content. BP has been involved in drafting the manuscript or revising it critically for important intellectual content. AR had a major contributor in writing the manuscript. MG has been involved in drafting the manuscript or revising it critically for important intellectual content. All authors read and approved the final manuscript.

## References

[B1] Williamson WA Tronic BS Levitan N Webb-Johnson DC Shahian DM Ellis FH Jr Pulmonary venous infarction Chest 1992 102 937 940 10.1378/chest.102.3.937 1516426

[B2] Dye TE Saab SB Almond CH Watson L Sclerosing mediastinitis with occlusion of pulmonary veins: manifestation and management J Thorac Cardiovasc Surg 1977 74 137 141 875431

[B3] Katzenstein AL Mazur MT Pulmonary infarct: an unusual manifestation of fibrosing mediastinitis Chest 1980 77 521 524 10.1378/chest.77.4.521 7357976

[B4] Stevens LH Hormuth DA Schmidt PE Atkins S Fehrenbacher JW Left atrial myxoma: pulmonary infarction caused by pulmonary venous occlusion Ann Thorac Surg 1987 43 215 217 3813712 10.1016/s0003-4975(10)60401-8

[B5] Julio AM Pulmonary vein thrombosis presenting as myocardial infarction Chest 2006 344S

[B6] Mario JG Leonardo R Pieter V Pulmonary vein thrombosis and peripheral embolization Chest 1996 109 846 847 10.1378/chest.109.3.846 8617103

[B7] Sarsam MA Yonan NA Beton D McMaster D Deiraniya AK Early pulmonary vein thrombosis after single lung transplantation J Heart Lung Transplant 1993 12 17 19 8443196

[B8] Nam HK Carlos AR Bruce KS Pulmonary vein thrombosis Chest 1993 104 624 625 10.1378/chest.104.2.624 8339662

[B9] McIlroy DR Sesto AC Buckland MR Pulmonary vein thrombosis, lung transplantation and intraoperative transesophageal echocardiography J Cardiothorac Vasc Anesth 2006 20 5 712 715 10.1053/j.jvca.2005.12.003 17094181

[B10] Selvidge SDD Gavant ML Idiphatic pulmonary vein thrombosis: detection by CT and MR imaging AJR 1999 172 1639 1641 10350306 10.2214/ajr.172.6.10350306

[B11] Dore R Alerci M D'Andrea F Di Giulio G De Agostini A Volpato G Intracardiac extension of lung cancer via pulmonary veins: CT diagnosis J Comput Assist Tomogr 1988 12 565 568 10.1097/00004728-198807000-00004 3392255

[B12] Shuman RL Primary pulmonary sarcoma with left atrial extension via left superior pulmonary vein. En bloc resection and radical pneumonectomy on cardiopulmonary bypass J Thorac Cardiovasc Surg 1984 88 189 192 6748712

[B13] Urschel HC Jr Razzuk MA Netto GJ Disiere J Chung SY Sclerosing mediastinitis: improved management with histoplasmosis titer and ketoconazole Ann Thorac Surg 1990 50 2 215 221 2383106 10.1016/0003-4975(90)90737-q

[B14] Garcia MJ Rodriguez L Vandervoort P Pulmonary vein thrombosis and peripheral embolization Chest 1996 109 846 847 10.1378/chest.109.3.846 8617103

[B15] Kim NH Roldan CA Shively BK Pulmonary vein thrombosis Chest 1993 104 624 626 10.1378/chest.104.2.624 8339662

